# Development of appropriate fatty acid formulations to raise the contractility of constructed myocardial tissues

**DOI:** 10.1016/j.reth.2022.09.006

**Published:** 2022-09-29

**Authors:** Azumi Yoshida, Waki Sekine, Jun Homma, Hidekazu Sekine, Yu Yamasaki Itoyama, Daisuke Sasaki, Katsuhisa Matsuura, Eiji Kobayashi, Tatsuya Shimizu

**Affiliations:** a Institute of Advanced Biomedical Engineering and Science, Tokyo Women's Medical University, Tokyo, Japan; b Department of Kidney Regenerative Medicine, The Jikei University, Tokyo, Japan

**Keywords:** Energy production, Fatty acid, β-oxidation, Culture medium, Cardiomyocyte, ATP, Adenosine triphosphate, DMEM, Dulbecco's Modified Eagle's Medium, DMEM-Hanks, DMEM containing Hanks' salts, DMEM-HG, DMEM containing 4500 mg/L glucose, DMEM-LG, DMEM containing 1000 mg/L glucose, FAD, Flavin adenine dinucleotide, FA-free, Medium without fatty acids, FA-mix, Medium containing a mixture of fatty acids, FBS, Fetal bovine serum, HBSS, Hanks' Balanced Salt Solution, hiPSC-CM, Human induced pluripotent stem cell-derived cardiomyocyte, NAD, Nicotinamide adenine dinucleotide, OCR, Oxygen consumption rate, P/S, Penicillin-streptomycin, rat-CM, Neonatal rat primary cardiomyocyte, TR-F, Time-resolved fluorescence

## Abstract

**Introduction:**

Heart disease is a major cause of mortality worldwide, and the annual number of deaths due to heart disease has increased in recent years. Although heart failure is usually managed with medicines, the ultimate treatment for end-stage disease is heart transplantation or an artificial heart. However, the use of these surgical strategies is limited by issues such as thrombosis, rejection and donor shortages. Regenerative therapies, such as the transplantation of cultured cells and tissues constructed using tissue engineering techniques, are receiving great attention as possible alternative treatments for heart failure. Research is ongoing into the potential clinical use of cardiomyocytes derived from human induced pluripotent stem cells (hiPSC-CMs). However, the energy-producing capacity of cardiomyocytes maintained under previous culture conditions is lower than that of adult primary cardiomyocytes due to immaturity and a reliance on glucose metabolism. Therefore, the aims of this study were to compare the types of fatty acids metabolized between cardiomyocytes in culture and heart cells *in vivo* and investigate whether the addition of fatty acids to the culture medium affected energy production by cardiomyocytes.

**Methods:**

A fatty acid-containing medium was developed based on an analysis of fatty acid consumption by rat primary cardiomyocytes (rat-CMs), and the effects of this medium on adenosine triphosphate (ATP) production were investigated through bioluminescence imaging of luciferase-expressing rat-CMs. Next, the fatty acid content of the medium was further adjusted based on analyses of fatty acid utilization by porcine hearts and hiPSC-CMs. Oxygen consumption analyses were performed to explore whether the fatty acid-containing medium induced hiPSC-CMs to switch from anaerobic metabolism to aerobic metabolism. Furthermore, the effects of the medium on contractile force generated by hiPSC-CM-derived tissue were evaluated.

**Results:**

Rat serum, human serum and porcine plasma contained similar types of fatty acid (oleic acid, stearic acid, linoleic acid, palmitic acid and arachidonic acid). The types of fatty acid consumed were also similar between rat-CMs, hiPSC-CMs and porcine heart. The addition of fatty acids to the culture medium increased the bioluminescence of luciferase-expressing rat-CMs (an indirect measure of ATP level), oxygen consumption by hiPSC-CMs, and contractile force generated by cardiac tissues constructed from hiPSC-CMs.

**Conclusions:**

hiPSC-CMs metabolize similar types of fatty acid to those consumed by rat-CMs and porcine hearts. Furthermore, the addition of these fatty acids to the culture medium increased energy production by rat-CMs and hiPSC-CMs and enhanced the contractility of myocardial tissue generated from hiPSC-CMs. These findings suggest that the addition of fatty acids to the culture medium stimulates aerobic energy production by cardiomyocytes through β-oxidation. Since cardiomyocytes cultured in standard media rely primarily on anaerobic glucose metabolism and remain in an immature state, further research is merited to establish whether the addition of fatty acids to the culture medium would improve the energy-producing capacity and maturity of hiPSC-CMs and cardiac tissue constructed from these cells. It is possible that optimizing the metabolism of cultured cardiomyocytes, which require high energy production to sustain their contractile function, will improve the properties of hiPSC-CM-derived tissue, allowing it to be better utilized for disease modeling, drug screening and regenerative therapies for heart failure.

## 1 Introduction

Heart disease is one of the main causes of mortality worldwide, and the annual number of deaths due to heart disease has increased in recent years [1]. Although heart failure is mainly managed with medicines, the ultimate treatment for severe disease is heart transplantation or an artificial heart [2]. However, these surgical treatments are associated with limitations such as thrombosis, rejection and donor shortages.

Regenerative therapies are currently being developed as potential alternative treatments for heart failure. One method that has been applied clinically is the transplantation of cultured cells directly into damaged tissue to stimulate repair via paracrine mechanisms [3], but the therapeutic effects of this technique are limited. Therefore, there has been great interest in the development of regenerative therapies based on the transplantation of bioengineered tissues [4]. We have developed a tissue engineering technology that allows the construction of three-dimensional tissues from cell sheets [[5], [6], [7]]. We have successfully constructed thick tissues by stacking cell sheets, and we have fabricated three-dimensional planar and annular myocardial tissues on vascular beds with arteries and veins that can be anastomosed to host vessels [[8], [9], [10]]. In addition, transplanted annular myocardial tissue was shown to generate pressure changes within the vena cava or the aorta of the rat [11,12]. Our recent studies have focused on the potential clinical applications of cardiomyocytes derived from human induced pluripotent stem cells (hiPSC-CMs). However, hiPSC-CMs are immature in structure and function when compared to primary cardiomyocytes [[13], [14], [15]], and tissues produced from hiPSC-CMs are immature in comparison to adult human myocardial tissue [16,17]. The main role of the heart to pump blood around the body requires mature cardiomyocytes with adequate contractile function. Therefore, recent studies have explored whether the addition of additive factors such as growth factors and fatty acids to the culture medium and three-dimensional culture methods can improve the maturation of hiPSC-CMs [[18], [19], [20]].

The culture of cardiomyocytes was first performed about 45 years ago, and M−199 and Dulbecco's modified eagle medium (DMEM) are commonly used culture media [21]. Although these media are thought to contain all the basic nutrients required for cell growth such as amino acids and vitamins, cardiomyocytes cultured in these media exhibit changes over time such as a decrease in cell number and the development of a rounded and shortened cell morphology [22]. These limitations of standard culture media have led to various attempts to improve the technique for cardiomyocyte culture. For example, the addition of fatty acids to the culture medium has been reported to enhance the maturation of cardiomyocytes [[23], [24], [25]]. Adenosine triphosphate (ATP) synthesis in the mitochondria is driven by a proton gradient generated by the electron transport chain, which involves the oxidation of flavin adenine dinucleotide (FAD) and nicotinamide adenine dinucleotide (NAD). The reduced FAD and NAD required for oxidative phosphorylation are generated from glucose metabolism via the glycolytic and tricarboxylic acid (TCA) cycles and from fatty acid metabolism via β-oxidation and the TCA cycle. The maximum amount of ATP synthesized from 1 mol glucose is 36 mol, whereas the synthesis of ATP from fatty acids is more efficient. For example, about 120 mol ATP can be synthesized from 1 mol stearic acid (C18:0; a saturated fatty acid). The glycolytic system is active in immature hiPSC-CMs and human fetal cardiomyocytes, but lactate oxidation and fatty acid oxidation become active in human cardiomyocytes during the neonatal period after birth. Notably, β-oxidation of fatty acids is responsible for about 50%–70% of energy production in healthy, mature cardiomyocytes [14,15,18,[26], [27], [28]]. Fatty acids are also metabolized in normal dog hearts and improve both mitochondrial function and oxygen consumption rate (OCR) [29].

We hypothesized that culturing cardiomyocytes in the presence of fatty acids would induce a switch from anaerobic glucose metabolism to aerobic fatty acid metabolism and thereby enhance the maturity and contractility of the cells as well as the functionality of myocardial tissue generated from hiPSC-CMs. Therefore, the aims of this study were to characterize the fatty acids metabolized by various types of cardiomyocyte and evaluate whether the addition of fatty acids to the culture medium enhanced ATP synthesis and oxygen consumption by cardiomyocytes as well as contractile force in cardiac tissues constructed from hiPSC-CMs.

## 2 Methods

All animal experiments were approved by the Ethics Committee for Animal Experimentation of Tokyo Women's Medical University and performed according to the Guidelines of Tokyo Women's Medical University on Animal Use. All animals were housed in individual cages with free access to food and water and maintained at constant room temperature and humidity under a 12-h light cycle.

### 2.1 Culture of neonatal rat primary cardiomyocytes (rat-CMs)

Hearts were removed from LEW/CrlCrlj (Charles River Laboratories Japan, Kanagawa, Japan) or LEW-Tg (Rosa-luc)11Jmsk (Jichi Medical University, Tochigi, Japan) neonatal rats aged 0–3 days old and washed three times with Hanks' Balanced Salt Solution (HBSS; Fujifilm Wako Pure Chemical, Tokyo, Japan) [[30], [31], [32]]. The hearts were minced into pieces less than 2 mm in size, and 7–8 pieces of tissue were placed into a gentleMACS C tube and processed in a gentleMACS dissociator (Miltenyi Biotec, Bergisch Gladbach, Germany). Cardiomyocytes were collected using a Neonatal Heart Dissociation Kit (Milteny Biotec) and selected using a Neonatal Cardiomyocyte Isolation Kit (Milteny Biotec). The number of cardiomyocytes obtained was counted. The cells were seeded on culture dishes coated with fetal bovine serum (FBS; Thermo Fisher Scientific, Tokyo, Japan) and cultured under specific conditions for each experiment.

### 2.2 Differentiation and purification of hiPSC-CMs

201B7 hiPSCs (Riken, Tsukuba, Japan), which contained the puromycin resistance gene under the control of the mouse α-myosin heavy chain promoter and the neomycin resistance gene under the control of the rex-1 promoter, were differentiated into hiPSC-CMs using the method described by Matsuura et al. [[33], [34], [35]]. The differentiated cardiomyocytes were purified by culture in DMEM containing 10% FBS (Thermo Fisher Scientific) and 2.5 ng/mL puromycin (Sigma–Aldrich Japan K.K, Tokyo, Japan) for 4 days.

### 2.3 Bioluminescence imaging of luciferase-expressing rat-CMs

Changes in cardiomyocyte ATP levels were evaluated indirectly through bioluminescence imaging of luciferase-expressing rat-CMs. LEW-Tg (Rosa-luc)11Jmsk rat-CMs (3 × 10^4^ cells/dish) were seeded on 3.5-cm-diameter culture dishes (day 0) and cultured at 37 °C in an atmosphere containing 5% CO_2_. DMEM containing 4500 mg/L glucose (DMEM-HG; Fujifilm Wako Pure Chemical) and supplemented with 10% FBS (Thermo Fisher Scientific) and 1% penicillin-streptomycin (P/S; Fujifilm Wako Pure Chemical) was used for the first 5 days of culture, and the medium was changed on day 3. The medium was replaced with test medium on day 5–6, and bioluminescence imaging was performed (LV200 LuminoView system; Olympus, Tokyo, Japan) to observe the time-dependent changes in the luciferase luminescence of each cell [36]. The two types of test medium used were FA-free medium, which consisted of glucose-free DMEM (Fujifilm Wako Pure Chemical) containing 25 μM L-carnitine (Sigma–Aldrich), and FA-mix medium, which consisted of glucose-free DMEM containing 25 μM L-carnitine and a mixture of fatty acids (oleic acid, stearic acid, myristic acid, linoleic acid, palmitic acid and arachidonic acid; 5 μM each; Sigma–Aldrich) dissolved in Tween 80 (Sigma–Aldrich). For all samples, the medium was changed to 1 mL FA-free medium containing 50 μL luciferin (Biosynth, Postfach, Switzerland) just before the start of imaging. The medium was then replaced with the appropriate test medium (FA-free or FA-mix) 10 min after the start of imaging. Imaging was performed during 2-min periods separated by 3-min intervals for a total of 1 h. This experiment was performed three times using cardiomyocytes grown in primary culture on different days, and each measurement was performed in triplicate.

### 2.4 Analysis of fatty acid consumption by rat-CMs and hiPSC-CMs

LEW/CrlCrlj rat-CMs or hiPSC-CMs (1 × 10^6^ cells/dish) were seeded on 3.5-cm-diameter culture dishes (day 0) and cultured at 37 °C in an atmosphere containing 5% CO_2_. The medium used from day 0 to day 5 was DMEM-HG (Fujifilm Wako Pure Chemical), 10% FBS (Thermo Fisher Scientific) and 1% P/S (Fujifilm Wako Pure Chemical), and the medium was changed on day 3. The medium was replaced on day 5 with 100% rat serum (Kohjin Bio, Saitama, Japan) for rat-CMs and 100% human serum (Kohjin Bio) for hiPSC-CMs. Samples of serum used as the culture medium were obtained before and 24 h after cell culture, and the lipid profile of each sample was obtained using gas chromatography. Fatty acid consumption was calculated by comparing the serum lipid profiles before and 24 h after culture. This experiment was performed three times using rat-CMs grown in primary culture on different days and hiPSC-CMs differentiated on different days, and each measurement was performed in duplicate.

### 2.5 Analysis of fatty acid consumption by porcine heart

Landrace pigs (Sanesu Breeding, Chiba, Japan) weighing about 15 kg were anesthetized using 40 μg/kg medetomidine hydrochloride (Domitor; Nippon Zenyaku Kogyo, Tokyo, Japan) and 0.25 mg/kg midazolam (Dormicum; Astellas Pharma, Tokyo, Japan) for induction and 2.5% sevoflurane inhalation anesthetic solution (Mylan; Pfizer, Tokyo, Japan) for maintenance. Blood samples from a coronary artery and the coronary sinus were collected into separate heparin tubes using a catheter, and the plasma was obtained by centrifugation at 3000 rpm for 10 min. The lipid profile of each plasma sample was obtained by gas chromatography, and fatty acid consumption was determined as the difference in content between the coronary arterial sample and coronary sinus sample. This experiment was performed on three different pigs, and each measurement was made in duplicate.

### 2.6 Analysis of oxygen consumption by hiPSC-CMs

HiPSC-CMs were seeded (8 × 10^4^ cells/well) on 96-well black plates (day 0) and cultured (37 °C, 5% CO_2_) in DMEM-HG (Fujifilm Wako Pure Chemical) containing 10% FBS (Thermo Fisher Scientific) and 1% P/S (Fujifilm Wako Pure Chemical). The medium was changed on day 3 to DMEM containing 1000 mg/L glucose (DMEM-LG; Fujifilm Wako Pure Chemical) and supplemented with 25 μM L-carnitine (Sigma–Aldrich) and a mixture of fatty acids (oleic acid, stearic acid, myristic acid, linoleic acid, linolenic acid, palmitic acid, palmitoleic acid and arachidonic acid; 50 μM each; Sigma–Aldrich). The medium was renewed on days 5 and 8 and then changed to test medium on day 10. The three types of test medium used were FA-free, which consisted of glucose-free DMEM (Fujifilm Wako Pure Chemical) containing 25 μM L-carnitine (Sigma–Aldrich) and 10 mg/L oxaloacetic acid (Sigma–Aldrich), FA-mix-5μM, which consisted of FA-free containing a mixture of fatty acids (oleic acid, stearic acid, myristic acid, linoleic acid, linolenic acid, palmitic acid, palmitoleic acid and arachidonic acid; 5 μM each; Sigma–Aldrich), and FA-mix-50μM, which consisted of FA-free containing 50 μM of each fatty acid. The next day (i.e., day 11), the amount of dissolved oxygen in the medium was measured using a MitoXpress Xtra-Oxygen Consumption Assay (H5-Method) Kit (Agilent Technologies, Santa Clara, CA, USA) in accordance with the kit instructions [37,38]. The measurement conditions were as follows: interval, 60 s; number of measurements, 30; total measurement time, 1 h. From the measurement results, the lifetime of the ratiometric time-resolved fluorescence (TR-F) and OCR were calculated. This experiment was performed six times using cardiomyocytes differentiated on different days, and each measurement was performed in triplicate.

### 2.7 Measurement of contractile force in myocardial tissue constructed from hiPSC-CMs

The contractile force of myocardial tissue constructed from hiPSC-CMs was measured using a modification of the method described by Sasaki et al. [39,40]. First, 25 mg/mL fibrinogen (Sigma–Aldrich), 160 U/mL factor XIII (CSL Behring, King of Prussia, PA, USA), 8 mM CaCl_2_ and 4 U/mL thrombin (Sigma–Aldrich) were mixed and poured into a mold that piled silicone sheets. The mixture was covered by an acrylic plate and left to stand for 30 min to solidify as a gel. Then, the gel was detached from the mold and stored at 37 °C in DMEM-HG containing 10% FBS, 1% P/S and 500 KIU/mL aprotinin (Fujifilm Wako Pure Chemical). On day 0, hiPSC-CMs (2 × 10^6^ cells/cm^2^) were seeded on the gel (12 mm × 12 mm) and cultured. On day 3, the myocardial tissue and gel were attached to a load cell (LVS-10GA; Kyowa Electronic Instruments, Tokyo, Japan) for measurement of contractile force. The lower handle of the gel was attached with a clip to a culture chamber constructed from acrylic plates, and the upper handle of the gel was attached to the sensor rod of the load cell via a hook. The sample was cultured in DMEM containing Hanks’ salts (DMEM-Hanks; Nissui Pharmaceutical Co., Ltd., Tokyo, Japan) containing 10% FBS, 1% P/S and 500 KIU/mL aprotinin. Any samples that did not generate contractile force on day 4 were excluded from further analysis. The medium was changed to test medium on day 4. Two types of test medium were used: FA-free medium, which consisted of DMEM-Hanks containing 10% FBS, 1% P/S, 500 KIU/mL aprotinin, 25 μM L-carnitine and 10 mg/L oxaloacetic acid, and FA-mix medium, which consisted of FA-free medium supplemented with a mixture of lipids (oleic acid, stearic acid, myristic acid, linoleic acid, linolenic acid, palmitic acid, palmitoleic acid and arachidonic acid; 50 μM each; Nissui Pharmaceutical Co., Ltd.). The medium was renewed on day 6. Contractile force was measured on day 7 during stimulation of the myocardial tissue with biphasic pulses (voltage, 10 V; duration, 10 msec; interval, 0.8 s) at 37 °C. The load cell was connected to a strain amplifier (DPM-712B; Kyowa Electronic Instruments), and the data were digitized and imported into a computer using a Power Lab 8/30 data acquisition system (ADInstruments, Bella Vista, Australia). This experiment was performed seven times using hiPSC-CMs that were differentiated on different days.

### 2.8 Statistical analysis

Data values are expressed as the mean ± standard error of the mean (SEM). Bioluminescence imaging data obtained from luciferase-expressing rat-CMs and lifetime data obtained from the analysis of oxygen consumption by hiPSC-CMs were analyzed by repeated-measures analysis of variance (ANOVA) and Holm's sequentially rejective Bonferroni procedure (post-hoc comparisons). OCR data were analyzed using one-way ANOVA and Dunnett's test (post-hoc comparisons). Contractile force data obtained from hiPSC-CM-derived myocardial tissue was analyzed using Student's t-test for paired comparisons. Statistical significance was considered at *P* < 0.05. R Software (R Foundation for Statistical Computing, Vienna, Austria) was used for the statistical analyses.

## 3 Results

### 3.1 Fatty acids commonly consumed by rat-CMs were identified

**Fig. 1:**
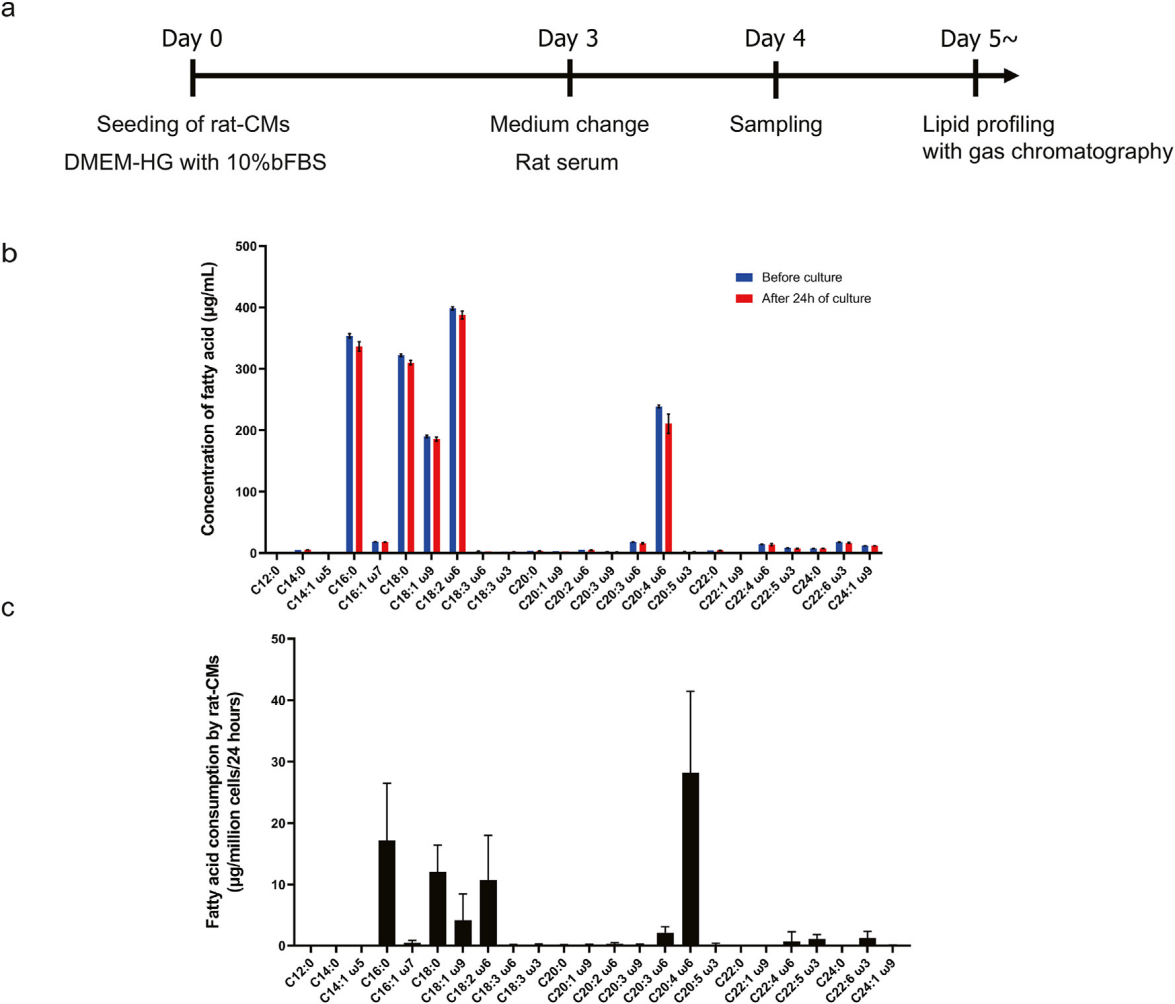
**Analysis of fatty acid consumption by rat-CMs.** (a) Protocol used to analyze fatty acid consumption by rat-CMs. Rat-CMs were seeded (day 0) and cultured for 3 days in DMEM-HG medium containing 10% FBS. The medium was changed to rat serum on day 3, and culture was continued for a further 24 h (day 4). Samples of the rat serum used as the medium were obtained before and 24 h after culture for assessment of the lipid profile using gas chromatography. (b) Concentrations of various fatty acids in rat serum before culture (blue) and 24 h after culture (red). Data are shown as mean ± SEM (*n* = 3 experiments in duplicate). C12:0, lauric acid; C14:0, myristic acid; C14:1, myristoleic acid; C16:0, palmitic acid; C16:1, palmitoleic acid; C18:0, stearic acid; C18:1, oleic acid; C18:2, linoleic acid; C18:3 ω6, ɤ-linoleic acid; C18:3 ω3, linolenic acid; C20:0, arachidic acid; C20:1, eicosenoic acid; C20:2, eicosadienoic acid; C20:3 ω9, 5-8-11 eicosatrienoic acid; C20:3 ω6, dihomo-ɤ-linolenic acid; C20:4, arachidonic acid; C20:5, eicosapentaenoic acid; C22:0, behenic acid; C22:1, erucic acid; C22:4, docosatetraenoic acid; C22:5, docosapentaenoic acid; C24:0, lignoceric acid; C22:6, docosahexaenoic acid; C24:1, nervonic acid. (c) Fatty acid consumption by rat-CMs. Each value is calculated as the difference between the fatty acid content of the serum before culture and the fatty acid content of the serum 24 h after culture. Data are shown as mean ± SEM (*n* = 3 experiments in duplicate). Abbreviations are as in (b).

Analysis of the fatty acid content of rat serum (Fig. 1a) revealed that linoleic acid was most abundant followed by palmitic acid, stearic acid, arachidonic acid and oleic acid (Fig. 1b). The main fatty acids consumed by rat-CMs (Fig. 1c) were arachidonic acid (a fall in medium concentration of 28.2 ± 13.3 μg/mL), palmitic acid (17.2 ± 9.3 μg/mL), stearic acid (12.1 ± 4.4 μg/mL), linoleic acid (10.7 ± 7.3 μg/mL) and oleic acid (4.2 ± 4.3 μg/mL).

### 3.2 Addition of fatty acids to the culture medium increased the levels of ATP in rat-CMs

**Fig. 2:**
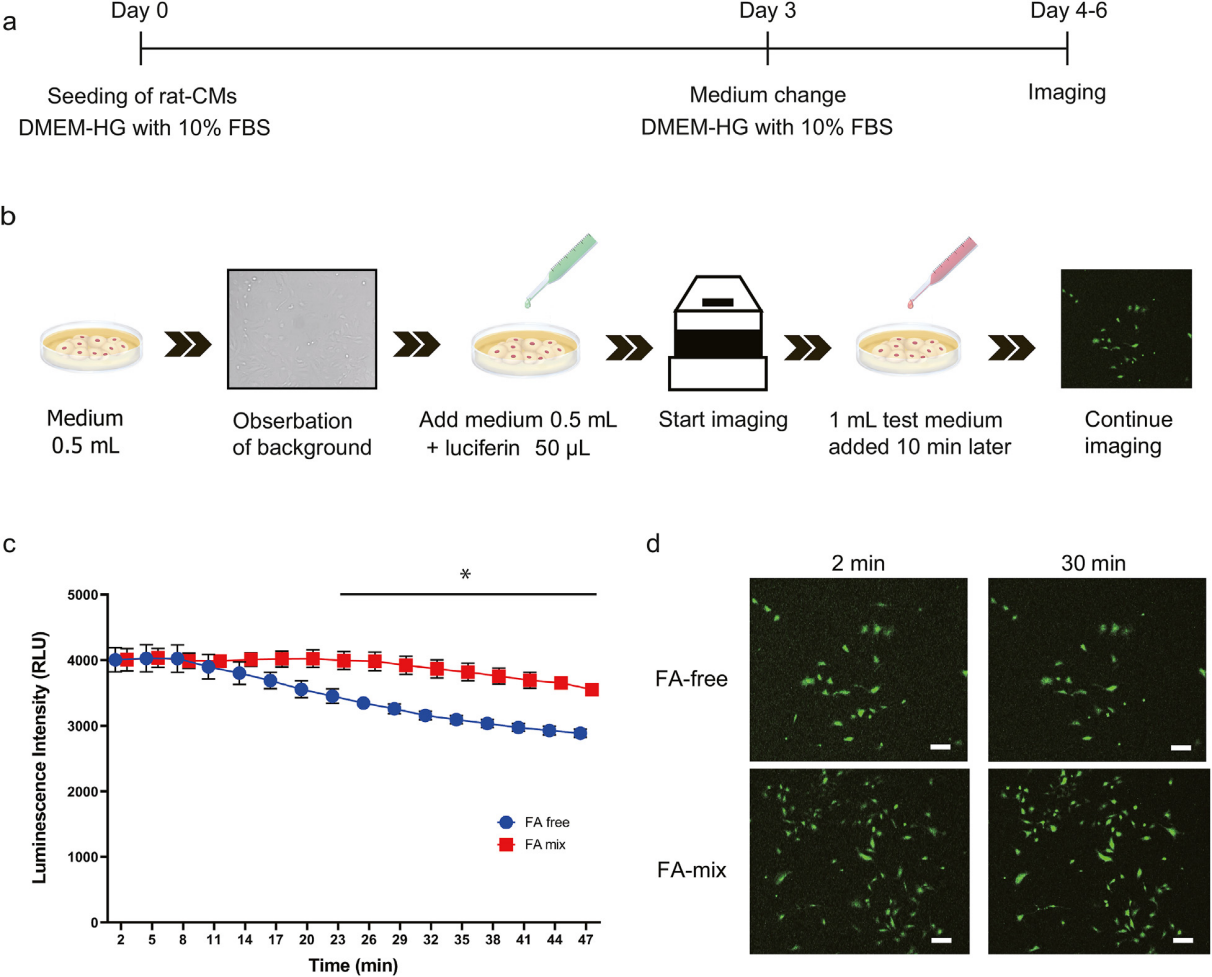
**Bioluminescence imaging of luciferase-expressing rat-CMs.** (a) Protocol used for bioluminescence imaging of luciferase-expressing rat-CMs. Rat-CMs were seeded (day 0) and cultured for 3 days in DMEM-HG medium containing 10% FBS. The medium was changed on day 3. Imaging was performed on days 4–6. (b) Imaging method used. First, medium without nutrients (0.5 mL) was added, and the background level was recorded. Next, medium without nutrients (0.5 mL) and luciferin (50 μL) were added, and imaging was started. Test medium (1 mL) was added 10 min later. Imaging was performed in 2-min blocks at 3-min intervals for a total of 1 h. (c) Changes in luminescence intensity over time (FA-free group, blue; FA-mix group, red). Data are presented as mean ± SEM (*n* = 3 experiments in triplicate). *∗P* < 0.05 (Holm's sequentially rejective Bonferroni test). (d) Representative images of luminescent cells obtained at 2 min and 30 min. Scale bar = 200 μm.

The cellular levels of ATP were evaluated indirectly through bioluminescence imaging of luciferase-expressing rat-CMs treated with luciferin (Fig. 2a and b). Luminescence is generated when luciferin is oxidized by luciferase, which is an ATP-dependent enzyme; hence, a higher level of luminescence in the rat-CMs was considered to reflect a higher level of intracellular ATP. The changes in luminescence intensity over time are shown in Fig. 2c. The luminescence intensity was comparable between the FA-mix and FA-free groups during the first 10 min of imaging. However, the luminescence intensity in the FA-free group started to decrease at about 13 min and fell from a maximal level of 4028.3 ± 206.7 at 5 min to 288.3 ± 60.6 at 47 min. By contrast, the luminescence intensity in the FA-mix group exhibited a smaller decline, decreasing from a maximal value of 4035.3 ± 143.4 at 5 min after the addition of fatty acids to 3552.5 ± 77.2 at 47 min. Notably, there was a significant difference between the two groups in the luminescence intensity between 23 min and 47 min (*P < 0.05*, Holm's sequentially rejective Bonferroni test). Representative bioluminescence images obtained at 2 min and 30 min are presented in Fig. 2d.

### 3.3 Fatty acids commonly consumed in porcine heart were identified

**Fig. 3:**
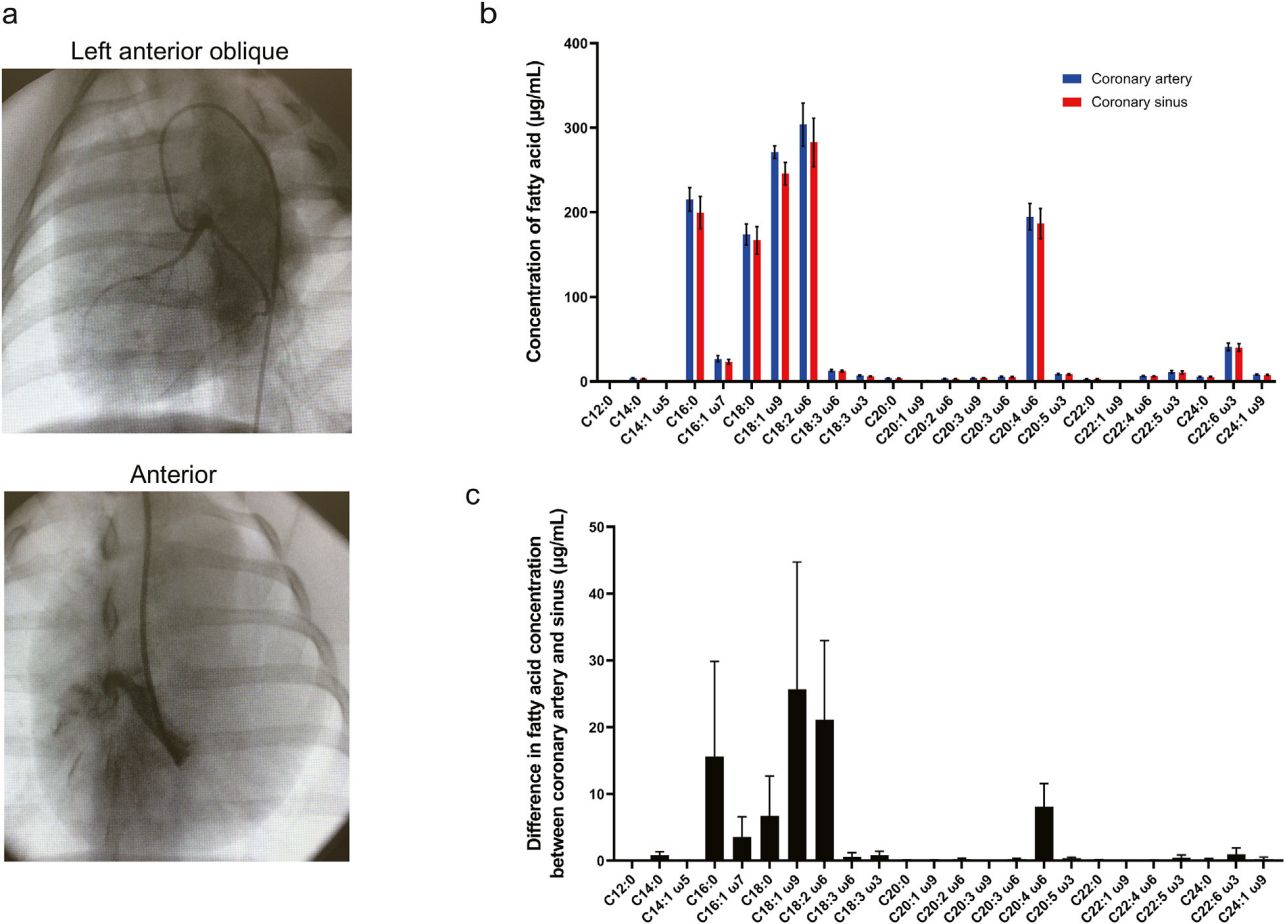
**Analysis of fatty acid consumption by porcine heart.** (a) Representative X-ray images illustrating blood sample collection from the porcine heart via a catheter. The left anterior oblique view shows the coronary arterial circulation filled with contrast, and the anterior view demonstrates the coronary sinus filled with contrast. (b) Concentrations of various fatty acids in plasma obtained from the coronary artery (red) and coronary sinus (blue) of the porcine heart. Data are shown as mean ± SEM (*n* = 4 experiments in duplicate). Abbreviations for fatty acids are the same as in Fig. 1(b). (c) Fatty acid consumption by the porcine heart evaluated as the difference in fatty acid content between the coronary artery and coronary sinus. Data are shown as mean ± SEM (n = 4 experiments in duplicate). Abbreviations are as in (b).

X-ray images illustrating the use of catheters to sample blood from the coronary arteries and coronary sinus of the porcine heart are presented in Fig. 3a. The most abundant fatty acids in coronary arterial blood and coronary sinus blood from the pig heart were linoleic acid, oleic acid, palmitic acid, arachidonic acid and stearic acid (Fig. 3b). Furthermore, the main fatty acids consumed by the porcine heart (Fig. 3c) were oleic acid (a difference in concentration between the coronary artery and coronary sinus of 25.7 ± 19.0 μg/mL), linoleic acid (21.1 ± 11.8 μg/mL), palmitic acid (15.6 ± 14.2 μg/mL), arachidonic acid (8.1 ± 3.5 μg/mL) and stearic acid (6.7 ± 5.9 μg/mL).

### 3.4 Fatty acids commonly consumed by hiPSC-CMs were identified

**Fig. 4:**
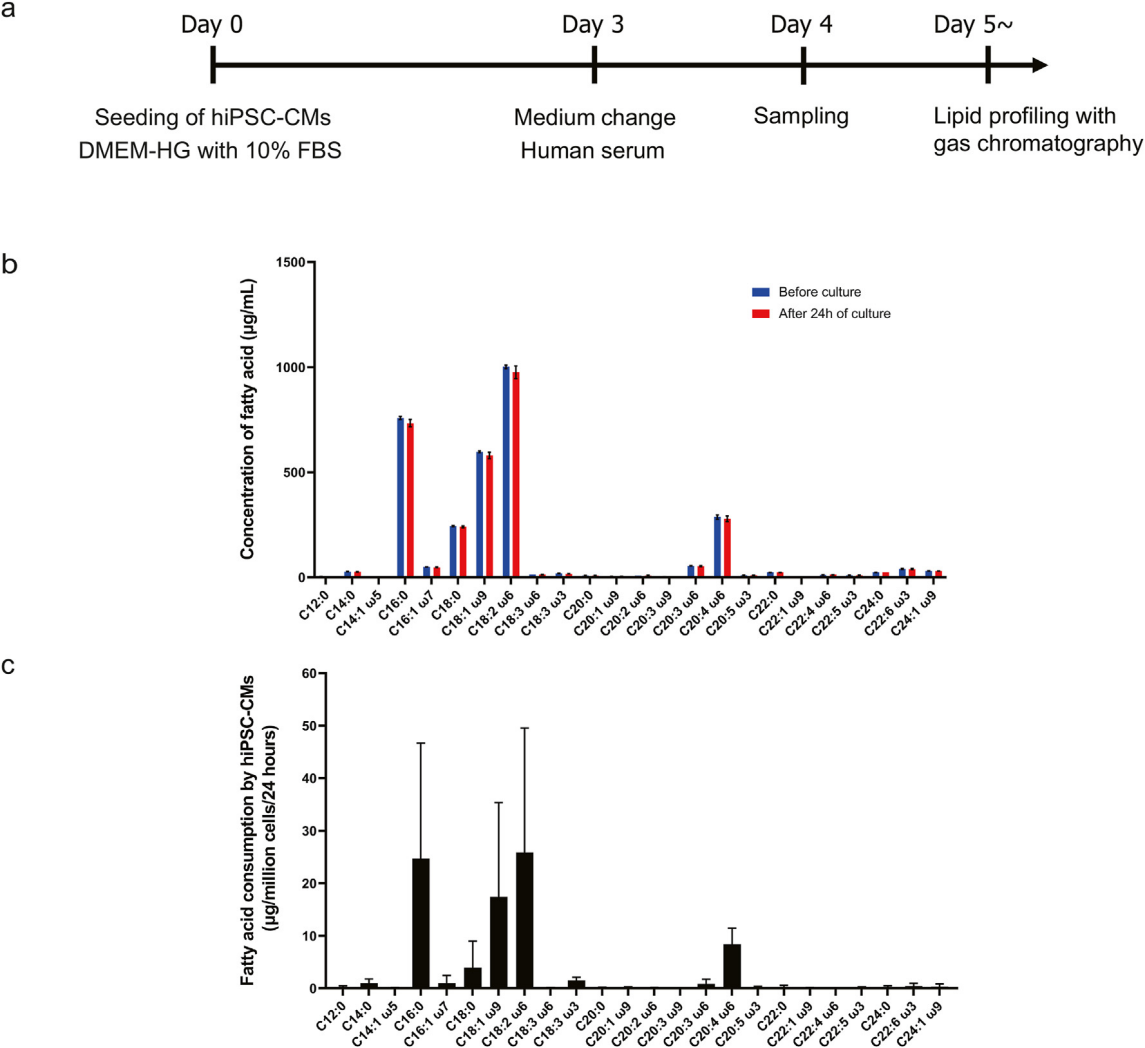
**Analysis of fatty acid consumption by hiPSC-CMs.** (a) Protocol used to analyze fatty acid consumption by hiPSC-CMs. The hiPSC-CMs were seeded (day 0) and cultured for 3 days in DMEM-HG medium containing 10% FBS. The medium was changed to human serum on day 3, and culture was continued for a further 24 h (day 4). Samples of the human serum used as the medium were obtained before and 24 h after culture for assessment of the lipid profile using gas chromatography. (b) Concentrations of various fatty acids in human serum before culture (blue) and 24 h after culture (red). Data are shown as mean ± SEM (n = 3 experiments in duplicate). Abbreviations for fatty acids are the same as in Fig. 1(b). (c) Fatty acid consumption by hiPSC-CMs. Each value is calculated as the difference between the fatty acid content of the serum before culture and the fatty acid content of the serum 24 h after culture. Data are shown as mean ± SEM (*n* = 3 experiments in duplicate). Abbreviations are as in (b).

Measurements of the fatty acid content of human serum (Fig. 4a) demonstrated that linoleic acid, palmitic acid, oleic acid, arachidonic acid and stearic acid were present at the highest concentrations (Fig. 4b). The main fatty acids consumed by HiPSC-CMs (Fig. 4c) were linoleic acid (a fall in medium concentration of 25.9 ± 23.6 μg/mL), palmitic acid (24.7 ± 22.0 μg/mL), oleic acid (17.5 ± 17.9 μg/mL), arachidonic acid (8.4 ± 3.1 μg/mL) and stearic acid (3.9 ± 5.1 μg/mL).

### 3.5 Addition of fatty acids to the culture medium increased the oxygen consumption of hiPSC-CMs

**Fig. 5:**
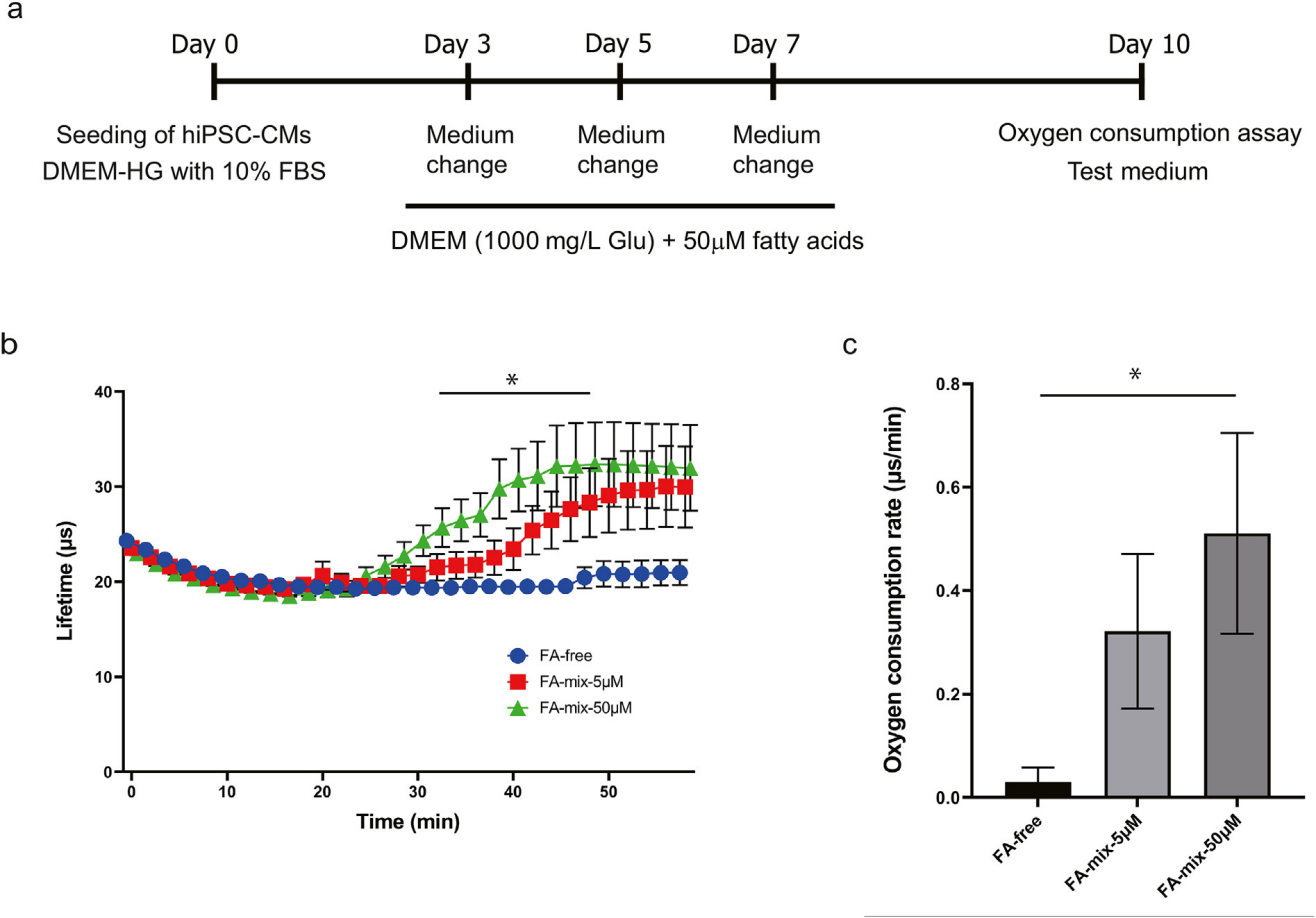
**Analysis of oxygen consumption by hiPSC-CMs.** (a) Protocol used to analyze oxygen consumption by hiPSC-CMs. The hiPSC-CMs were seeded (day 0) and cultured for 3 days in DMEM-HG medium containing 10% FBS. The medium was changed to DMEM containing 1000 mg/mL glucose and a mixture of fatty acids (50 μM each) on days 3, 5 and 7. Oxygen consumption was assessed on day 10 using a test medium. (b) Fluorescence lifetime values for the FA-free group (blue), FA-5μM group (red) and FA-50μM group (green). Data are presented as mean ± SEM (*n* = 6). ∗*P* < 0.05, FA-free group vs. FA-50μM group (Holm's sequentially rejective Bonferroni test). (c) OCR between 20 min and 50 min after starting the measurement. Data are presented as mean ± SEM (n = 6 experiments in triplicate). ∗*P* < 0.05, FA-free group vs. FA-50μM group (Dunnett's test).

Fig. 5a summarizes the protocol used for the oxygen consumption analyses. Fig. 5b shows the TR-F lifetime calculated from the oxygen consumption analyses. There was little or no change in oxygen consumption following the addition of FA-free, which contained no fatty acids. By contrast, the administration of FA-mix-5μM (which contained various fatty acids, each at a concentration of 5 μM) resulted in an increase in oxygen consumption from 20 min onwards, and a greater increase was observed following the administration of FA-mix-50μM (which contained 50 μM of each fatty acid). There was a significant difference between the FA-free and FA-50μM groups in the lifetime value between 30 min and 46 min (*P < 0.05*, Holm's sequentially rejective Bonferroni test). Fig. 5c shows the OCR between 20 min and 50 min after the start of the measurement. The OCR was 0.03 ± 0.03 for the FA-free group, 0.32 ± 0.15 for the FA-5μM group and 0.51 ± 0.19 for the FA-50μM group. Moreover, there was a significant difference in OCR between the FA-free and FA-50μM groups (*P < 0.05*, Dunnett's test).

### 3.6 Addition of fatty acids to the culture medium increased the contractile force of myocardial tissue constructed from hiPSC-CMs

**Fig. 6:**
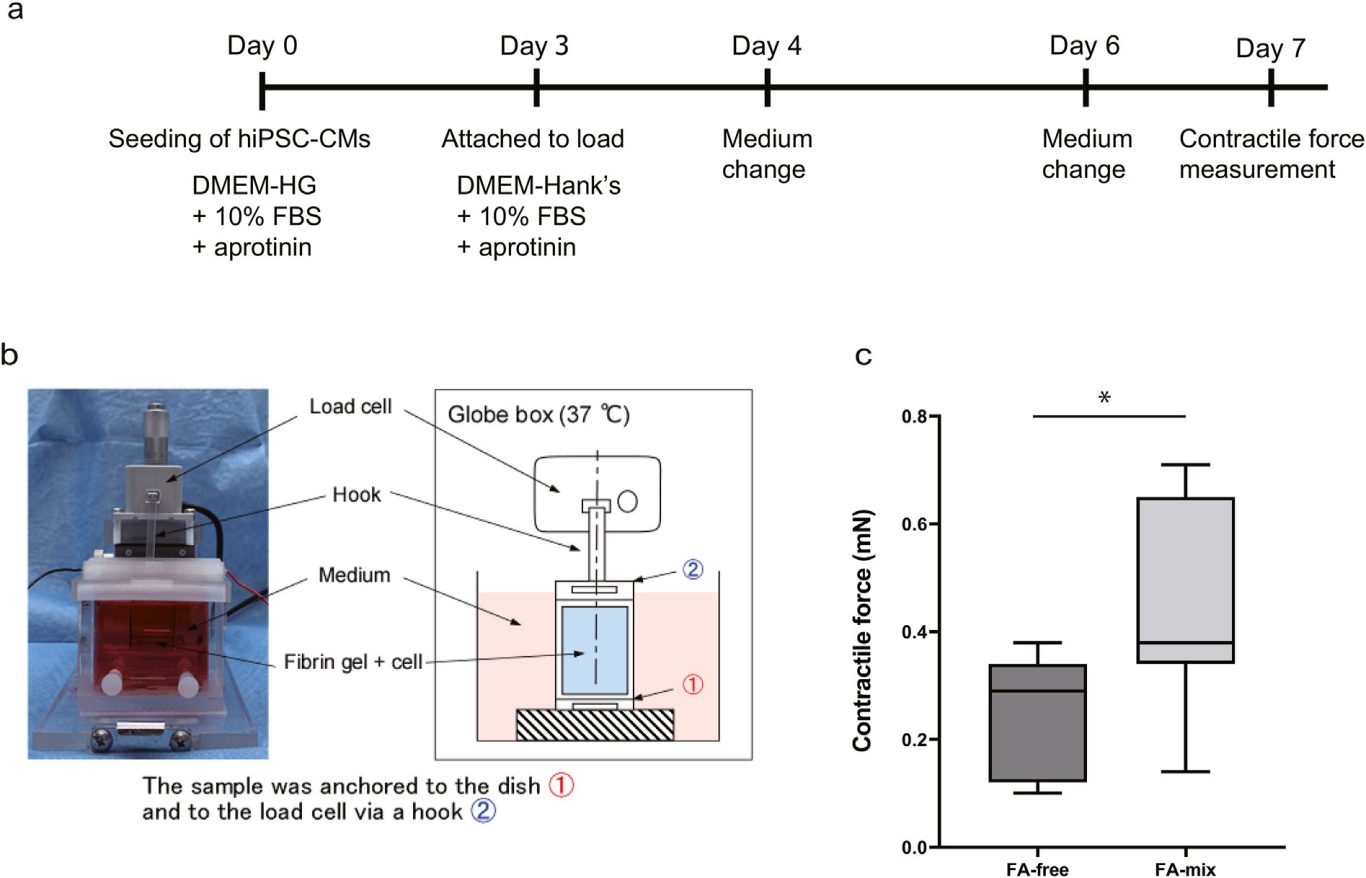
**Measurement of contractile force generated by myocardial tissue derived from hiPSC-CMs.** (a) Protocol used to measure the contractile force of myocardial tissues constructed from hiPSC-CMs. The hiPSC-CMs were seeded on a fibrin gel on day 0 and cultured for 3 days in DMEM-HG containing 10% FBS and aprotinin. The sample was attached to the load cell on day 3 and cultured for 24 h in DMEM-Hanks containing 10% FBS and aprotinin. The medium was changed to test medium on day 4, which was renewed on day 6, and contractile force was measured on day 7. (b) Photograph and schematic of the apparatus used to measure the contractile force generated by myocardial tissue. The gel containing the myocardial tissue was anchored at its lower aspect to the dish and at its upper aspect to the load cell (via a hook). (c) Contractile force generated by hiPSC-CM-derived myocardial tissues. The contractile force generated of FA-mix group and FA-free group was 0.44 ± 0.07 mN and 0.24 ± 0.04 mN (mean ± SEM), respectively. Box plots show summary statistics. Boxes indicate the 25–75% range; dividers indicate the median. The bottom edge of the whiskers indicates the minimum value and the top edge indicates the maximum value (*n* = 7). *∗P < 0.05* (paired t-test).

The minimum contraction force in the FA-free group was 0.10 mN and the maximum was 0.38 mN. On the other hand, the minimum value of contractility for the FA-mix group was 0.14 mN and the maximum value was 0.71 mN. Although there were differences depending on the cells used and the conditions of the tissue produced, the contractility of the samples in the FA-mix group was greater than that of the samples in the FA-free group each time in the samples within the same trial. As shown in Fig. 6, the contractile force generated by hiPSC-CM-derived myocardial tissue was significantly higher in the FA-mix group than in the FA-free group (0.44 ± 0.07 mN vs. 0.24 ± 0.04 mN, *P < 0.05*, paired t-test).

## 4 Discussion

The analysis of fatty acid consumption performed in the present study successfully identified the types of fatty acid used by rat cardiomyocytes, hiPSC-CMs and porcine hearts. Furthermore, it was shown that cultured cardiomyocytes were able to utilize fatty acids for energy production. Additionally, the availability of fatty acids led to an increase in the contractile force generated by myocardial tissue constructed from hiPSC-CMs.

We found that rat serum, human serum and porcine plasma contained similar types of fatty acid such as oleic acid, stearic acid, linoleic acid, palmitic acid and arachidonic acid. Furthermore, there were some similarities in the types of fatty acid consumed by rat-CMs, hiPSC-CMs and porcine hearts, although some differences between species were also noted. The main types of fatty acid consumed were particularly similar between porcine hearts and cultured hiPSC-CMs, which may reflect physiological and biochemical similarities between pigs and humans [41]. Our analysis also revealed that cardiomyocytes metabolized a wider variety of fatty acids than previously reported [23,24,42,43]. In this study, the fatty acids to be added to the culture medium were selected on the basis of changes in the amount of use. Fatty acids taken up by cells are activated to acyl CoA in the cytoplasm and then bound to carnitine, which enters the mitochondria and is used for β-oxidation. Therefore, In this study, carnitine, which is a carrier for fatty acids to enter the mitochondria, was also added together [44]. Furthermore, the experiment was conducted in a culture condition with1000 mg/L of glucose, close to the *in vivo* concentration, which may have further promoted fatty acid utilization. The addition of oxaloacetic acid, which binds to acetyl CoA generated by β-oxidation, also stimulated the TCA circuit to work, which is thought to have resulted in the production of more energy. On the other hand, fatty acids are also used for fatty acid biosynthesis in the cytoplasm and phospholipid biosynthesis, as well as β-oxidation in mitochondria. In this study, we believe that the addition of eight fatty acids, more than in previous studies, may have promoted efficient β-oxidation and energy production by improving mitochondrial function from various perspectives. Further analysis will be necessary to optimize the concentration and combination of fatty acids.

The luciferase-expressing rats used for luminescence imaging in this study have been shown to be capable of ATP quantification in room temperature perfusate and have also been used for imaging in tissues created from cardiomyocytes in vitro [31,32]. Thus, the intensity of the luminescence emitted by luciferase-expressing rat cardiomyocytes was considered to reflect the cellular level of ATP. Thus, the increase in luminescence intensity detected after the addition of fatty acids likely occurred due to the activation of energy production by the rat cardiomyocytes. Similarly, the inclusion of fatty acids in the medium led to a significant increase in oxygen consumption by hiPSC-CMs. This increase in oxygen consumption may have resulted from a switch from anaerobic metabolism to aerobic metabolism that enhanced energy production and consequently oxygen consumption. Our result would be consistent with the previous observation that an increase in free fatty acid uptake into the heart was associated with a 26% increase in muscle myocardial volume oxygen (an estimation of the amount of oxygen used by the heart) despite no change in cardiac function [29]. This effect may have been due to enhanced β-oxidation of fatty acids (i.e., aerobic metabolism) followed by active energy production by the respiratory chain. Interestingly, Huang et al. reported that fatty acids activated a strong, potentiodependent calcium current in cardiomyocytes that developed 10–20 min after the addition of fatty acids [45]. Our bioluminescence and oxygen consumption analyses also demonstrated changes that developed 10–20 min after the inclusion of fatty acids in the culture medium. The above results suggest that cardiomyocytes may take up fatty acids within 10–20 min of their addition and then enhance energy production in the mitochondria through β-oxidation of the fatty acids. This finding would be consistent with a previous report that cultured cardiomyocytes take up fatty acids within 20 min [23].

We suggest that the increase in oxygen consumption by cardiomyocytes following the addition of fatty acids occurred as a result of enhanced ATP synthesis by the electron transport chain. Specifically, β-oxidation of fatty acids in the mitochondria provides acetyl CoA for the TCA cycle. In turn, the TCA cycle generates FAD and NAD, which are utilized in the electron transport chain for ATP synthesis. However, energy production by immature cardiomyocytes mainly relies on glucose metabolism, and β-oxidation of fatty acids only becomes the major metabolic pathway after the cells have matured [14]. However, when cells are cultured in a standard medium containing a high concentration of glucose, the abundance of glucose may limit the opportunity for the cells to utilize fatty acids. In this study, the bioluminescence imaging experiments and oxygen consumption analyses were carried out using medium with a glucose concentration that was lower than that of standard culture media and similar to that of the blood *in vivo*, which may have promoted a shift to fatty acid-based metabolism. We suggest that the addition of fatty acids to a low-glucose culture medium can switch the metabolism of cardiomyocytes from the glycolytic system to β-oxidation of fatty acids, thereby increasing energy production.

The contractile force generated by myocardial tissue derived from hiPSC-CMs was increased nearly two-fold following the addition of fatty acids to the culture medium. As cardiomyocytes mature, there is a shift in metabolism from glycolysis to β-oxidation of fatty acids, and this is followed by structural maturation and a subsequent increase in contractility [15]. Our findings raise the possibility that culture in a medium supplemented with fatty acids not only switches the preferred metabolic pathway from glycolysis to β-oxidation of fatty acids but also enhances cardiomyocyte maturation and thereby increases contractility. Although we demonstrated that the provision of fatty acids caused an enhancement of contractility in the short-term, it will be necessary to examine whether this effect is maintained in the long term and whether it would be possible to further increase contractility to create tissue with functionality that is closer to that of adult myocardial tissue.

Although the addition of fatty acids to the culture medium may have promoted maturation of the cells and the efficiency of energy production, the cells remained immature in terms of their functionality and morphology relative to adult primary cardiomyocytes. In addition, the bioluminescence imaging and oxygen consumption analyses show that energy production exhibited an initial increase after the addition of fatty acids followed by a decrease after a certain amount of time. This may be due to a lack of luciferin added as a substrate, since the experimental system uses chemical reactions such as the luciferin-luciferase reaction, but it may also be due to a lack of fatty acids in the medium. It was notable that in the experiments measuring contractility, in which the ratio of the volume of medium to the number of cells was high, no decrease in contractility was observed for about 2 days after the medium was changed (data not shown). The concentrations of fatty acids added in this study (5 μM or 50 μM) were lower than those utilized in previous studies [19,20], which suggests that a depletion of fatty acids may have been responsible for the reduction in energy production observed in the bioluminescence imaging experiments and oxygen consumption analyses. To overcome this potential issue, we are currently developing methods to continuously and stably provide fatty acids to cells by increasing the concentration of fatty acids added, dissolving the fatty acids in alternative solvents (such as bovine serum albumin) that have fewer adverse effects on cells, and studying methods of adding fatty acids such as reflux systems. If the cells can be continuously supplied with the necessary fatty acids, as *in vivo*, it may be possible to mature them into cardiomyocytes that are structurally and functionally closer to those of the adult heart *in vivo*. If a method was developed to mature cardiomyocytes in culture, it could also be applied to the creation of highly contractile, three-dimensional, vascularized myocardial tissue using our scaffold-free tissue engineering technology [[5], [6], [7], [8], [9], [10], [11],^16^,^17^]. Since the contractile function of adult cardiomyocytes requires very high energy production, understanding the metabolic characteristics of hiPSC-CMs and developing ways to mature these cells will facilitate the creation of regenerative tissue for drug screening, disease modeling and heart failure therapy.

## 5 Conclusion

The present study has characterized the types of fatty acid consumed by rat cardiomyocytes, hiPSC-CMs and porcine hearts. Moreover, the findings confirmed that cultured cardiomyocytes and myocardial tissues can utilize fatty acids for energy production. Notably, it was shown that the addition of fatty acids to the culture medium not only enhanced energy production by cultured rat cardiomyocytes and hiPSC-CMs but also increased the contractile force generated by myocardial tissue constructed from hiPSC-CMs.

## Declaration of competing interest

Tatsuya Shimizu is a shareholder of CellSeed Inc. Katsuhisa Matsuura and Tatsuya Shimizu are the inventors of bioreactor systems. Tokyo Women's Medical University and received research funding from CellSeed Inc. and Nihon Kohden Corporation. The other authors have no potential conflicts of interest.
